# Small contact resistance and high-frequency operation of flexible low-voltage inverted coplanar organic transistors

**DOI:** 10.1038/s41467-019-09119-8

**Published:** 2019-03-08

**Authors:** James W. Borchert, Boyu Peng, Florian Letzkus, Joachim N. Burghartz, Paddy K. L. Chan, Karin Zojer, Sabine Ludwigs, Hagen Klauk

**Affiliations:** 1 Max Planck Institute for Solid State Research, Heisenbergstr. 1, Stuttgart, 70569 Germany; 2 Institute of Polymer Chemistry, University of Stuttgart, Pfaffenwaldring 55, Stuttgart, 70569 Germany; 3 Department of Mechanical Engineering, The University of Hong Kong, Pokfulam Road, Hong Kong SAR, China; 4 Institut für Mikroelektronik (IMS CHIPS), Allmandring 30a, Stuttgart, 70569 Germany; 5 Institute of Solid State Physics, NAWI Graz, Graz University of Technology, Petersgasse 16, 8010 Graz, Austria

## Abstract

The contact resistance in organic thin-film transistors (TFTs) is the limiting factor in the development of high-frequency organic TFTs. In devices fabricated in the inverted (bottom-gate) device architecture, staggered (top-contact) organic TFTs have usually shown or are predicted to show lower contact resistance than coplanar (bottom-contact) organic TFTs. However, through comparison of organic TFTs with different gate-dielectric thicknesses based on the small-molecule organic semiconductor 2,9-diphenyl-dinaphtho[2,3-*b*:2’,3’-*f*]thieno[3,2-*b*]thiophene, we show the potential for bottom-contact TFTs to have lower contact resistance than top-contact TFTs, provided the gate dielectric is sufficiently thin and an interface layer such as pentafluorobenzenethiol is used to treat the surface of the source and drain contacts. We demonstrate bottom-contact TFTs fabricated on flexible plastic substrates with record-low contact resistance (29 Ωcm), record subthreshold swing (62 mV/decade), and signal-propagation delays in 11-stage unipolar ring oscillators as short as 138 ns per stage, all at operating voltages of about 3 V.

## Abstract (web summary)

The widespread adoption of organic thin-film transistors (TFTs) in low-voltage high-frequency device applications is impeded by the contact resistance in the TFTs. Here, the authors report record-low contact resistance in bottom-gate, bottom-contact organic TFTs with an ultrathin gate dielectric.

## Introduction

To enable the adoption of organic thin-film transistors (TFT) in high-frequency device applications, the contact resistance must be reduced well below the smallest values reported to date^1–3^. The reason is that the contact resistance is a key limiting factor determining the transit frequency of organic TFTs^4,5^., and when the TFT dimensions are in the range required for megahertz operation at low voltages, the contact resistance is more limiting to the transit frequency than the intrinsic carrier mobility^2^. For example, achieving a transit frequency of 10 MHz in a TFT with a channel length of 1 µm and a total gate-to-contact overlap of 10 µm operating with voltages of 3 V requires the contact resistance to be smaller than 40 Ωcm, regardless of whether the intrinsic channel mobility is 10, 100, or 1000 cm^2^ V^−1^ s^−1^.^2^. In organic TFTs, the contact resistance is greatly affected not only by the choice of materials, but also by the choice of TFT architecture. In the case of the inverted (bottom-gate) architectures, the staggered (top-contact; TC) configuration has typically provided smaller contact resistance than the coplanar (bottom-contact; BC) configuration, even for devices comprising the same materials and layer thicknesses^6,7^. To date, the smallest contact resistances reported for TC and BC organic TFTs are 46.9 Ωcm^1^ and 80 Ωcm^8^, respectively. These and other experimental observations are in line with most device simulations that predict that TC organic TFTs would generally outperform BC organic TFTs due to lower contact resistance^7,9^. The smaller contact resistance of TC organic TFTs is ascribed primarily to the overlap between the contacts and a portion of the gate-induced carrier channel directly under the contacts, leading to more efficient charge injection^10–12^. Additionally, the electrical conductivity in the contact regions may be enhanced by metal clusters penetrating into the semiconductor layer upon deposition of the contact metal^13^, by contact doping^14^, and by the relatively large area for charge injection between the contact metal and the gate-induced carrier channel (current crowding)^10,15^.

In BC organic TFTs, the contact resistance is typically higher, since the gate field-assisted charge injection is weakened^9^. In addition, BC organic TFTs often exhibit a discontinuous coverage and poor thin-film morphology of the organic semiconductor layer along and across the edges of the contacts. Such a poor semiconductor morphology occurs, because the surface energy of the contact material usually differs profoundly from that of the gate dielectric^6^. Various approaches have been implemented to improve the wetting behavior of organic semiconductors on metal contacts, such as ozone exposure^8^, oxygen-plasma treatment^16^, and chemisorbed molecular monolayers^17^. Chemisorbed molecular monolayers show particular promise for the modification of both the gate dielectric and the contacts, because such modifications are area-selective, reproducible and when chosen carefully do not adversely affect subsequent processing steps^18–22^. For metal contacts, thiol monolayers can improve the morphology of the organic semiconductor layer above the contacts and across the contact edges and can lower the injection barrier by tuning the work function of the metal contacts by a few hundred milli-electronvolts^23–27^. The most utilized and effective molecule to date for improving the charge injection in p-channel organic TFTs is pentafluorobenzenethiol (PFBT)^26,28^. The increase in the work function is caused by the large interface dipole created by the high density of fluorine atoms in the PFBT monolayer^29^. The successful use of PFBT to improve the performance of p-channel BC organic TFTs has motivated investigations into other molecules capable of forming monolayers, particularly those with a large number of fluorine atoms to induce a work function shift beyond that obtained with PFBT^24^. Despite these efforts to improve the contact-semiconductor interface of BC organic TFTs, their contact resistance is still largely inferior to that of the best TC organic TFTs^1^.

However, recent drift-diffusion-based simulations performed by Zojer et al. predict that BC organic TFTs may exhibit lower contact resistances than otherwise equivalent TC organic TFTs^11,30^, provided the energy barrier between the source contact and the organic semiconductor is sufficiently low and the gate dielectric is sufficiently thin. Given the importance of the contact resistance for the dynamic TFT performance^1,2^, this is a potentially critical finding, but an experimental study to confirm the impact of the gate-dielectric thickness on the contact resistance has to our knowledge not yet been performed, although investigations into the effects of the gate-dielectric thickness on other organic-TFT-performance parameters are abundant^31–34^.

Here, we fabricated BC and TC organic TFTs with different thicknesses of aluminum oxide passivated with an alkylphosphonic acid self-assembled monolayer (SAM) as the gate dielectric. We used gold for the source and drain contacts, PFBT to treat the contacts of the BC TFTs, and the vacuum-deposited small-molecule semiconductor 2,9-diphenyl-dinaphtho-[2,3-b:2’,3’-f]thieno[3,2-b]thiophene (DPh-DNTT)^35–37^. DPh-DNTT has previously shown low contact resistance in TC TFTs^38^. We measured the contact resistance using the transmission line method (TLM) and found that when the gate-dielectric thickness is sufficiently small, the contact resistance is indeed smaller in the BC TFTs than in the TC TFTs, supporting the prediction by Zojer et al.^30^. Further, we fabricated TFTs and circuits on flexible plastic substrates, utilizing an aluminum oxide/SAM hybrid gate dielectric with a thickness of 5.3 nm. For the TC TFTs, we measured a contact resistance of 56 Ωcm, very similar to the contact resistance reported by Yamamura et al. for this device architecture on a glass substrate^1^. For the BC TFTs, we obtained an even smaller contact resistance of 29 Ωcm, the smallest contact resistance reported to date for organic TFTs using a non-electrolyte gate dielectric^39^. Furthermore, the signal-propagation delay, measured in flexible 11-stage unipolar ring oscillators at a supply voltage of 3.7 V, is 178 ns per stage for the TC TFTs and 138 ns per stage for the BC TFTs, confirming the benefit of a small contact resistance for the dynamic transistor performance. These are the shortest signal-propagation delays reported to date for organic ring oscillators at a supply voltage of less than 50 V, and they represent a significant step towards the use of organic TFTs in flexible low-power electronics applications.

## Results

### Organic TFTs with different gate-dielectric thicknesses

**Fig. 1:**
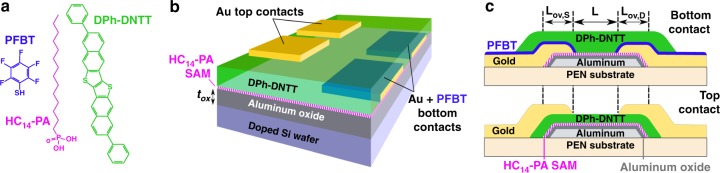
Organic thin-film transistors fabricated for this study. **a** Chemical structures of pentafluorobenzenethiol (PFBT) used to treat the surface of the gold bottom contacts, of *n*-tetradecylphosphonic acid used for the gate-dielectric self-assembled monolayer (SAM), and of the organic semiconductor 2,9-diphenyl-dinaphtho-[2,3-b:2’,3’-f]thieno[3,2-b]thiophene (DPh-DNTT). **b** Schematic cross-section of bottom-contact and top-contact organic TFTs fabricated on silicon substrates to study the relation between the gate-oxide thickness and the contact resistance. **c** Schematic cross-section of bottom-contact and top-contact organic TFTs fabricated on PEN substrates to evaluate the contact resistance and the dynamic performance of TFTs and circuits on flexible plastic substrates

To maintain the highest possible degree of comparability between the performance of the BC and TC TFTs, multiple measures were taken to render the devices in the two architectures as equivalent as possible. This is necessary to be able to base the comparison of contact resistance on controlled assumptions. As a first measure to maintain comparability, we fabricated the TFTs in close proximity to each other on a common substrate, utilizing the same gate-oxide layer and the same semiconductor layer (Fig. 1). With this measure in place, we only directly compare those contact resistances that are extracted from TFTs fabricated on the common substrate, i.e., from TFTs sharing the same gate-dielectric thicknesses. Secondly, we settled on a common nominal thickness of the vacuum-deposited DPh-DNTT (Fig. 1a) layer of 20 nm for all TFTs in this study. This is the optimum semiconductor-layer thickness that we have previously identified for TC organic TFTs based on DPh-DNTT^37^. Note that the semiconductor-layer thickness is relevant for the contact resistance in two ways. In TC organic TFTs, a larger thickness is expected to increase the contact resistance due to the poor vertical carrier transport and the increase in trap-state density with increasing thickness of small-molecular-semiconductor layers^1,12,40–42^. The intrinsic carrier mobility in the charge accumulation region, in turn, partially determines the bulk resistivity component of the contact resistance in the context of current crowding^12^. The importance of this effect will depend on the particular semiconductor and the TFT architecture, since a high intrinsic mobility in the accumulation region can mitigate the larger space-charge limitations on injection in the staggered configuration^12,43^. As a third measure, we omitted the use of contact doping for the TC TFTs, because it would compromise the comparability to the BC organic TFTs. Contact doping has shown the potential to reduce space-charge limitations of the contact resistance in TC TFTs, possibly by reducing the width of the Schottky barrier at the contact-semiconductor interface and by filling trap states in the semiconductor region directly under the contacts and in parts of the channel region adjacent to the contacts, with charges generated by the dopant^20,44,45^. Finally, PFBT (Fig. 1a) was used to modify the gold bottom contacts, which primarily serves to maintain similar semiconductor morphology across the contact-to-channel interface^26,46^. The use of PFBT has an additional benefit in terms of the charge-injection barrier at the contact-semiconductor interface of the BC TFTs^24^.

Unfortunately, the PFBT treatment is not applicable to TC TFTs due to the buried contact-semiconductor interface, but it is nonetheless an acceptable measure for the BC TFTs in this comparative study for three reasons. First, due to the high surface energy of the gold contacts, the bottom contacts must be modified in some way to enable a fair comparison of contact resistance between these architectures at all. Second, the very large contact areas in the TFTs on the silicon substrates ensures that the largest contributor to the contact resistance in the TC TFTs is the bulk resistance under the contacts and not the interface resistance^10^. Finally, an Ohmic contact resistance is required to justify the use of TLM to evaluate the contact resistance^47^. To that end, gold contacts were used for the TC TFTs, since the work function of gold (5.0 eV) is close to the HOMO energy level of DPh-DNTT (5.3 eV)^48^, and because penetration of gold clusters into small-molecule semiconductor films has been shown to reduce the dipole barrier that can otherwise form at the contact-semiconductor interface^49^. In the BC TFTs, Ohmic contact resistance can be realized by treating the gold contacts with an interface layer, which can increase the work function. This is accomplished with PFBT, since the large dipole moment pointing towards the –SH bonding group increases the effective work function of the gold to around 5.4 eV^24,50^.

### Semiconductor thin-film morphology

**Fig. 2:**
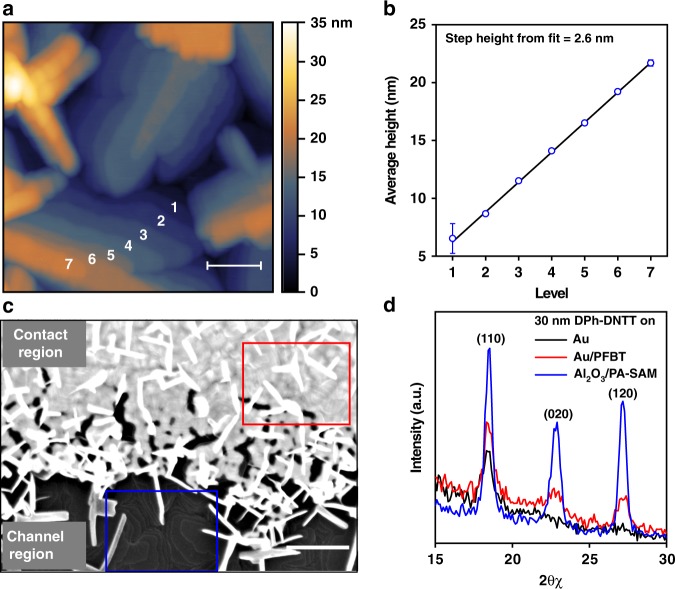
Morphological characterization of vacuum-deposited DPh-DNTT films. **a** AFM topography image of a DPh-DNTT layer formed on the Al_2_O_3_/SAM gate dielectric. Individual terrace levels are indicated with numbers 1–7. The scale bar corresponds to 100 nm. **b** Analysis of the height differences between the terraces. The step height of 2.6 nm corresponds closely to the length of a DPh-DNTT molecule, indicating that the molecules in each layer are oriented approximately upright with respect to the gate-dielectric surface. The data points and the error bars were obtained by local averaging of the terrace height over a large number of locations on each terrace in the AFM height image. **c** SEM image of the contact edge of a DPh-DNTT bottom-contact TFT with PFBT-treated gold contacts. The terrace morphology of the DPh-DNTT film is distinguishable on the contact surface near the contact edge (red box) and in the channel region (blue box). The scale bar corresponds to 200 nm. **d** Grazing incidence X-ray diffraction measurements on 30-nm-thick DPh-DNTT films on surfaces consisting of Au (black), PFBT-treated Au (red), and atomic-layer-deposited Al_2_O_3_ passivated with *n-*tetradecylphosphonic acid (PA-SAM, blue). The (110), (020), and (120) peaks are clearly distinguished in both of the latter two cases, signifying in-plane π–π stacking^48^, while on bare Au only the (110) peak is present

Atomic force microscopy (AFM) and scanning electron microscopy (SEM) analyses of the vacuum-deposited DPh-DNTT films show that the semiconductor morphology is very similar on all substrates, regardless of the type of substrate (silicon or PEN), the method by which the gate oxide was formed (atomic-layer deposition or plasma oxidation), and the gate-oxide thickness. The DPh-DNTT films show the characteristic terrace-like structure (Fig. 2a–c) that has been observed for this and other small-molecule semiconductors^37,38^ and is indicative of in-plane π–π stacking. This is additionally confirmed by grazing incidence X-ray diffraction (GIXRD) measurements of the DPh-DNTT films deposited onto the dielectric surface (see Fig. 2d)^48^. In the particular case of the BC TFTs, SEM, and GIXRD reveal that the treatment of the gold contacts with PFBT promotes the extension of the terrace-like DPh-DNTT film morphology in the channel region along and across the source and drain contact edges (Fig. 2c). Conversely, the GIXRD spectrum from DPh-DNTT deposited onto bare gold shows only the (110) peak, indicating poor in-plane π–π stacking on the gold surface.

### Gate-dielectric thickness and contact resistance

**Fig. 3:**
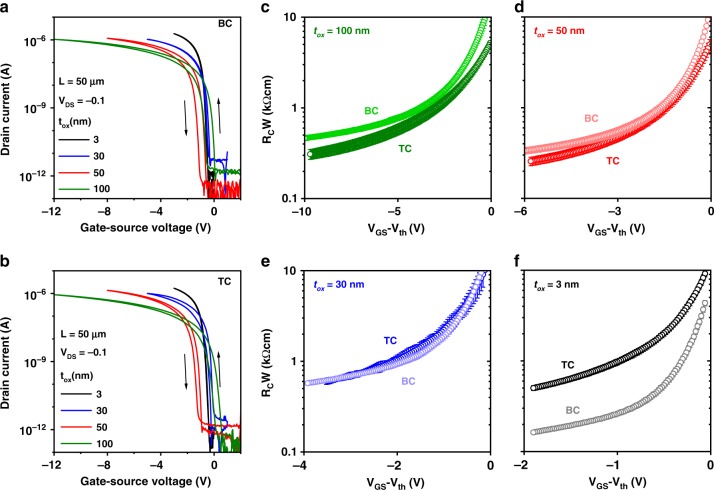
Bottom-contact and top-contact DPh-DNTT TFTs with different gate-dielectric thicknesses. The TFTs have gate-oxide thicknesses (*t*_*ox*_) of 3, 30, 50, and 100 nm, a channel length of 50 µm, and a channel width of 200 µm. **a**, **b** Transfer characteristics measured in the linear regime of operation (*V*_*DS*_ = −0.1 V). **c**–**f** Channel-width-normalized contact resistance (*R*_*C*_*W*) of TFTs with channel lengths ranging from 6 to 50 µm extracted in the linear regime of operation (*V*_*DS*_ = −0.1 V) and plotted as a function of the gate-overdrive voltage (*V*_*GS*_*−V*_*th*_), showing the influence of the gate-dielectric thickness on the contact resistance. For the smallest gate-oxide thickness of 3 nm, the contact resistance of the bottom-contact (BC) TFTs is smaller than that of the top-contact (TC) TFTs

Representative measured transfer characteristics of the BC and TC TFTs fabricated on silicon substrates are shown in Fig. 3a, b. Regardless of gate-dielectric thickness and the TFT architecture, all TFTs have a threshold voltage close to zero, a subthreshold swing smaller than 200 mV/decade, and an on/off current ratio of about 10^6^. To quantify the contact resistance of the TFTs, we employed the widely used transmission line method (TLM)^47^. The TLM assumes that the total device resistance (*R*) is the sum of a channel-length-independent and Ohmic contact resistance (*R*_*C*_) comprising both the source and drain contact resistances and a channel resistance proportional to the channel length (*L*)^47^. The channel-width-normalized resistance (*RW*) is determined at a drain-source voltage (*V*_*DS*_) as close to zero as possible (here: *V*_*DS*_ = −0.1 V) for a set of TFTs with channel lengths ranging from 6 to 50 µm. Based on the above-mentioned assumptions, the TLM deduces, for each desired gate-overdrive voltage (*V*_*GS*_*−V*_*th*_), a channel-width-normalized contact resistance (*R*_*C*_*W*) from the linear dependence on *L* of *RW*. In our TLM results, the linear fits of *RW* versus *L* for all gate-oxide thicknesses and gate-overdrive voltages are of good quality, with adjusted R² values > 0.9 (Supplementary Figure 1). Regardless of the device architecture and the gate-oxide thickness (*t*_*ox*_), *R*_*C*_*W* is always smaller than 1 kΩcm at sufficiently large *V*_*GS*_*−V*_*th*_, indicative of a small injection barrier.

The contact resistances obtained for the two device architectures and the four gate-oxide thicknesses are plotted in Fig. 3c–f as a function of the gate-overdrive voltage. As can bee seen, the difference between the contact resistances of the bottom-contact and top-contact TFTs depends on the gate-oxide thickness. It is intriguing to compare these results to a prediction obtained by two-dimensional drift-diffusion simulations, according to which the difference between the contact resistances of BC and TC TFTs will scale with the gate-dielectric thickness^30^. Indeed, when the gate-oxide thickness is large (≥50 nm), the contact resistance of our TC TFTs is smaller than that of our BC TFTs over the entire range of *V*_*GS*_*−V*_*th*_. At a medium gate-oxide thickness (30 nm), the contact resistances are approximately equal. When the gate-oxide thickness is sufficiently small (3 nm), the contact resistance in the BC TFTs is significantly smaller than in the TC TFTs over the entire range of gate-overdrive voltages. This confirms that there is an opportunity for BC TFTs to outperform TC TFTs in terms of contact resistance, provided the gate dielectric is sufficiently thin and the injection barrier at the contact-semiconductor interface is small.

In addition to the observation that the difference between the contact resistances of the BC and TC TFTs depends on the gate-oxide thickness, there are also noticeable differences in the shapes of the curves showing the contact resistance as a function of the gate-overdrive voltage (Fig. 3c–f): At small *V*_*GS*_*−V*_*th*_, the slope of the *R*_*C*_*W* vs. *V*_*GS*_−*V*_*th*_ curve is always steeper for the BC than for the TC TFTs, whereas at large *V*_*GS*_*−V*_*th*_, the slope is nearly the same when *t*_*ox*_ = 100, 50, or 30 nm (note the logarithmic scaling of *R*_*C*_*W* in Fig. 3c–f). For *t*_*ox*_ = 3 nm, the slope is always smaller for the BC TFTs and is also the smallest overall. We postulate that these features are related to the dependence of the contact resistance on two factors whose relative contributions depend on the TFT architecture: the geometry-specific electric-field distribution at the contact interface and the bulk resistance of the semiconductor layer between the contacts and the channel. As mentioned previously, the bulk resistance contributes significantly more strongly to the contact resistance in TC than in BC TFTs^12,51^. In TC TFTs, the bulk resistance is primarily modulated by the thickness of the semiconductor layer, i.e., a greater semiconductor-layer thickness results in a larger access resistance. This would likely result in a larger gate-oxide thickness below which BC TFTs have lower contact resistance than otherwise comparable TC TFTs. For BC TFTs, on the other hand, the contact resistance is primarily determined by the hole-injection barrier at the contact-semiconductor interface and the presence of space charges in the semiconductor^43^. Therefore, the contact resistance depends strongly on the electric field and less so on the semiconductor-layer thickness^30,52,53^. In addition, the contact resistance in BC TFTs has been shown to be dependent on the carrier mobility of the semiconductor. If the mobility is small and the charge injection efficient, space charges may build up that will inhibit carrier flow away from the region directly adjacent to the source contact, especially at small gate-source voltages^40,43^. Hence, for the BC TFTs with *t*_*ox*_ = 3 nm, the contact resistance and its dependence on the gate-overdrive voltage are reduced overall.

### Low-voltage bottom-contact and top-contact TFTs on flexible PEN substrates

**Fig. 4:**
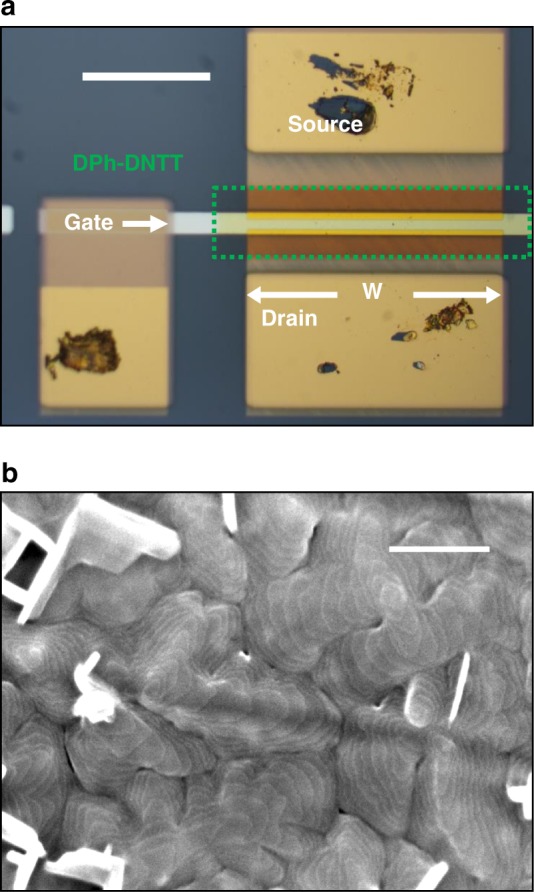
DPh-DNTT TFTs fabricated on flexible PEN substrates. **a** Optical microscopy image of a bottom-contact TFT with a channel length of 8 µm, a total gate-to-contact overlap (sum of the gate-to-source and gate-to-drain overlaps) of 10 µm, and a channel width of 200 µm on a flexible PEN substrate. The scale bar corresponds to 100 µm. **b** SEM image of the DPh-DNTT film in the channel region of a bottom-contact TFT on the same substrate. The terrace-like morphology of the organic semiconductor film is clearly distinguished and similar to that formed on the silicon substrates with atomic-layer-deposited aluminum oxide. The scale bar corresponds to 200 nm

**Fig. 5:**
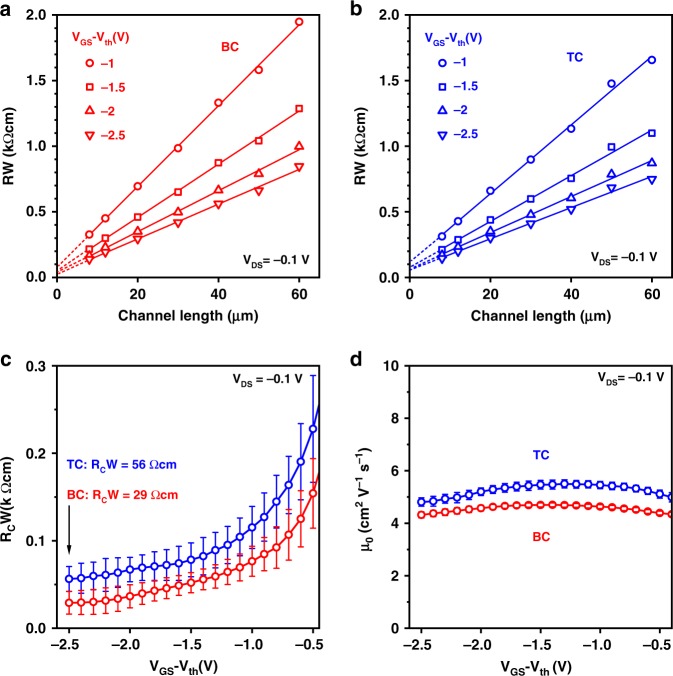
TLM analysis of bottom-contact and top-contact DPh-DNTT TFTs fabricated on flexible PEN substrates. The TFTs have a gate-dielectric thickness of 5.3 nm, channel lengths ranging from 8 to 60 µm, and a channel width of 200 µm. **a**, **b** Linear fits to the total width-normalized resistance (*RW*) at selected gate-overdrive voltages (*V*_*GS*_−*V*_*th*_). **c** Width-normalized contact resistance (*R*_*C*_*W*) plotted as a function of the gate-overdrive voltage. The error bars correspond to the standard error from the linear regression at each gate-overdrive voltage. **d** Intrinsic channel mobility (*µ*_*0*_) plotted as a function of the gate-overdrive voltage

We next show that the small contact resistance of bottom-contact DPh-DNTT TFTs with very thin gate dielectrics is evident also in TFTs fabricated on flexible plastic substrates (schematic shown in Fig. 1c). A photograph of a BC TFT fabricated on a PEN substrate is shown in Fig. 4a. An SEM image of the channel region of a DPh-DNTT TFT on a PEN substrate (Fig. 4b) indicates that the thin-film morphology of the DPh-DNTT films on the PEN substrates on which the plasma-grown aluminum oxide is used for the gate dielectric is similar to the thin-film morphology on the silicon substrates with the atomic-layer-deposited aluminum oxide. This is to be expected, since both oxide surfaces were treated in an identical manner with an *n*-tetradecylphosphonic acid SAM. For the extraction of the contact resistance we performed TLM analysis on TFTs with channel lengths ranging from 8 to 60 µm, a total gate-to-contact overlap (sum of the gate-to-source and gate-to-drain overlaps, *L*_*ov,total*_) of 10 µm, and a channel width of 200 µm. The results of the TLM measurements are shown in Fig. 5 and summarized in Table 1. The quality of the linear fits to the data in the *RW* vs. *L* graphs is again excellent, with adjusted R² ≥ 0.99. At a gate-overdrive voltage of −2.5 V, the TC TFTs have a channel-width-normalized contact resistance of 56 Ωcm, very similar to the record-low contact resistance reported by Yamamura et al. for TFTs fabricated in the same device architecture^1^. Despite the lower intrinsic channel mobility (Fig. 5d), the BC TFTs have an even smaller contact resistance of 29 Ωcm, which is to our knowledge the smallest contact resistance reported to date for organic transistors fabricated in the coplanar device architecture. For comparison, Stadlober et al. reported a contact resistance of 80 Ωcm for bottom-gate, bottom-contact pentacene TFTs in which the surface of the gold source and drain contacts had been exposed to ultraviolet radiation and ozone in order to induce a favorable pentacene morphology on the contacts^8^. Braga et al. reported the smallest contact resistance yet reported for organic TFTs of any architecture in electrolyte-gated top-gate staggered poly(3-hexylthiophene) TFTs in which strong doping of the semiconductor from the electrolyte resulted in a very small contact resistance of 1 Ωcm^39^.

**Table 1:** Summary of the results of the TLM measurements performed on top-contact and bottom-contact DPh-DNTT TFTs fabricated on flexible PEN substrates

Device architecture	*R*_*C*_*W* (Ωcm) at *V*_*GS*_−*V*_*th*_ = −2.5 *V*	*L*_*T*_ *(µm)* at *V*_*GS*_−*V*_*th*_ = −2.5 V	*µ*_*0*_ (cm^2^ V^−1^ s^−1^)	*L*_*1/2*_ (µm)
TC	56 ± 14	2.4 ± 0.6	5.7 ± 0.1	4.3 ± 0.2
BC	29 ± 13	1.1 ± 0.5	4.9 ± 0.1	2.6 ± 0.2

The intrinsic channel mobility (µ_0_) and the channel length at which the effective carrier mobility is half the intrinsic channel mobility (L_1/2_) were extracted from the fits in Supplementary Figure 2c

In addition to the contact resistance, the TLM analysis also yields the transfer length (*L*_*T*_). In staggered TFTs, *L*_*T*_ is the contact length over which 63% of the charge-carrier exchange occurs between the contact and the semiconductor^12,54^. For our TC TFTs, the transfer length is 2.4 µm, which is significantly smaller than the gate-to-source and gate-to-drain overlaps (*L*_*ov,S*_ and *L*_*ov,D*_), signifying that the injection is not limited by the contact area^55^. Despite the fact that the physical meaning of the transfer length in BC TFTs has so far not been elucidated, we still report it in Table 1 for comparison. The effective carrier mobility (*µ*_*eff*_) extracted from the transfer curves in the linear regime of operation (*V*_*DS*_ = −0.1 V) is less affected by the contact resistance in TFTs with long channel lengths (Supplementary Figure 2c). The intrinsic channel mobility (*µ*_*0*_) and the channel length at which the effective carrier mobility is half the intrinsic channel mobility (*L*_*1/2*_) were extracted using Equation 1 from ref. ^37^.

**Fig. 6:**
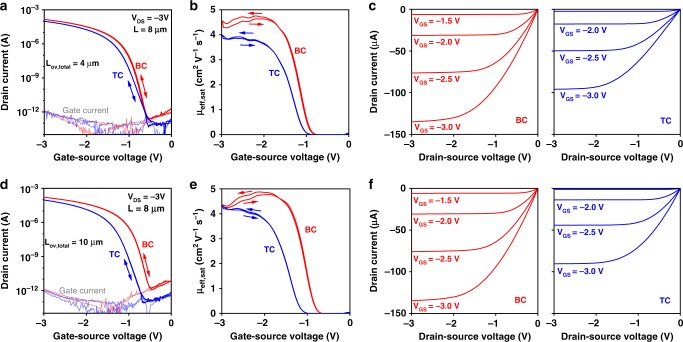
Static electrical characteristics of bottom-contact and top-contact DPh-DNTT TFTs on flexible PEN substrates. The TFTs have a gate-dielectric thickness of 5.3 nm, a channel length of 8 µm, a channel width of 200 µm, and a total gate-to-contact overlap of 4 µm (**a**–**c**) or 10 µm (**d**–**f**). **a**, **d** Transfer characteristics measured in the saturation regime (*V*_*DS*_ *=* *−*3 V). **b**, **e** Effective carrier mobility extracted from the transfer characteristics in the saturation regime (*V*_*DS*_ = -3 V). **c**, **f** Output characteristics of the same TFTs

The transfer and output characteristics of DPh-DNTT TFTs with a channel length of 8 µm and total gate-to-contact overlaps (*L*_*ov,total*_ = *L*_*ov,S*_ *+* *L*_*ov,D*_) of 4 and 10 µm are shown in Fig. 6. All TFTs have threshold voltages of about −1 V and on/off current ratios, here defined as the ratio between the drain current at V_GS_ = −3V and the lowest drain current measured in the transfer curve, between 10^8^ and 10^9^. The smaller contact resistance of the BC TFTs results in a larger effective carrier mobility compared to the TC TFTs (see Fig. 6 and Table 2).

**Table 2:** Summary of the static performance of top-contact and bottom-contact DPh-DNTT TFTs shown in Fig. 6, having a channel length of 8 µm and a channel width of 200 µm

Device architecture	L_ov,total_ (µm)	µ_eff,sat_ (cm² V^−1^ s^−1^)	SS (mV/dec)	On/off ratio
TC	4	3.9	94	10^9^
TC	10	4.2	92	10^9^
BC	4	4.6	62–64	10^9^
BC	10	4.4	68	10^8^

*V*_*DS*_ −3 V for all measurements

All TFTs have subthreshold swings (*SS*) smaller than 100 mV/decade, but those of the BC TFTs are notably smaller (62–68 mV/decade) than those of the TC TFTs (92–94 mV/decade). For the BC TFT with the smallest subthreshold swing, we have extracted the exact subthreshold swing using two different methods: once by fitting an exponential function to the data over a range of 200 mV in the subthreshold regime and once by point-wise derivation of the measured transfer curves (Supplementary Figure 3). Depending on the method and the applied drain-source voltage, the subthreshold swing is between 62 and 64 mV/decade. To our knowledge, this is the smallest subthreshold swing reported to date for organic TFTs, regardless of device architecture, gate dielectric, and semiconductor^56–59^. The observation that the BC TFTs have a notably smaller subthreshold swing than the TC TFTs suggests that the subthreshold swing is affected not only by the charge-trap density at the interface between the gate dielectric and the semiconductor layer (which is nominally identical in the two device architectures), but also by the charge-trap density in the semiconductor volume that separates the top contacts from the gate-induced carrier channel which the carriers have to traverse in the TC TFTs.

### Flexible low-voltage bottom-contact TFTs with small channel lengths

**Fig. 7:**
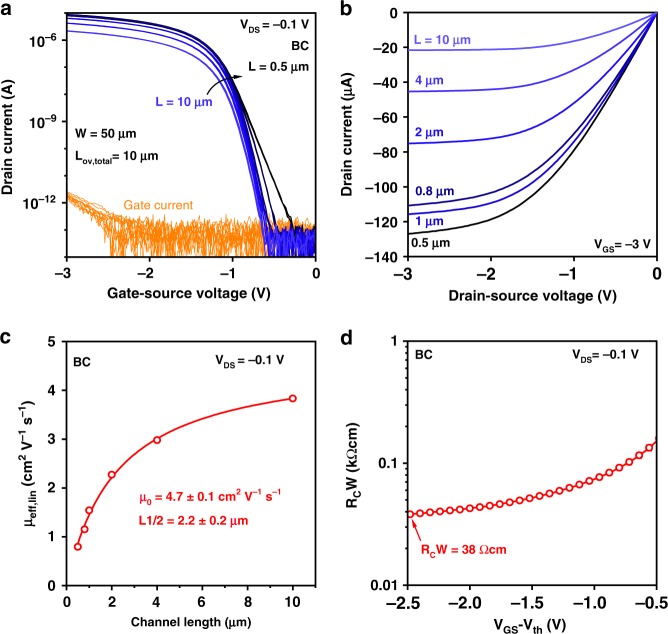
Short-channel bottom-contact DPh-DNTT TFTs on flexible PEN substrates. The TFTs have a gate-dielectric thickness of 5.3 nm, channel lengths ranging from 0.5 to 10 µm, a channel width of 50 µm, and a total gate-to-contact overlap of 10 µm. **a** Transfer characteristics measured in the linear regime of operation (*V*_*DS*_ = −0.1 V). **b** Output characteristics of the same TFTs measured at a gate-source voltage of −3 V. **c** Effective carrier mobility (*µ*_*eff*_) plotted as a function of the channel length. The error bars correspond to the standard error from the linear regression at each gate-overdrive voltage. **d** Channel-width-normalized contact resistance (*R*_*C*_*W*) plotted as a function of the gate-overdrive voltage (*V*_*GS*_−*V*_*th*_)

Often when the channel length is reduced, short-channel effects, such as drain-induced barrier lowering and increased off-state drain current, can become more prominent^55^. To investigate whether our flexible BC TFTs show any of these effects, we have fabricated short-channel bottom-contact DPh-DNTT TFTs on PEN substrates. These TFTs have a channel length ranging from 0.5 µm to 10 µm, a channel width of 50 µm, and a total gate-to-contact overlap of 10 µm. Regardless of the channel length, all TFTs have an on/off current ratio of at least 10^8^ (Fig. 7a), and the output curves do not show any noticeable non-linearity at small drain-source voltages that would indicate Schottky contacts (Fig. 7b)^60^. All TFTs with a channel length of at least 0.8 µm show effective carrier mobilities above 1 cm^2 ^V^−1^ s^−1^ (Fig. 7c). The TLM analysis again shows a low contact resistance of 38 Ωcm at a gate-overdrive voltage of −2.5 V (Fig. 7d, Supplementary Figure 4).

### Dynamic performance of flexible bottom-contact and top-contact TFTs

**Fig. 8:**
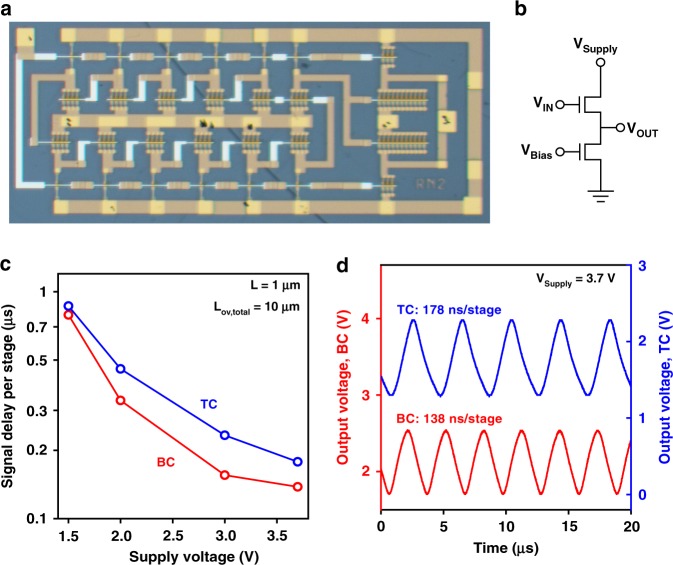
Dynamic performance of unipolar ring oscillators on flexible PEN substrates. **a** Photograph of an 11-stage unipolar ring oscillator based on bottom-contact DPh-DNTT TFTs with a channel length of 1 µm and a total gate-to-contact overlap of 10 µm. **b** Circuit diagram of the biased-load inverters implemented for the ring oscillators. **c** Signal-propagation delay per stage measured on ring oscillators based on top-contact(TC) and bottom-contact(BC) TFTs plotted as a function of the supply voltage. **d** Output signals measured at a supply voltage of 3.7 V, showing stage delays of 178 ns for the ring oscillator based on TC TFTs and 138 ns for the ring oscillator based on BC TFTs. Depending on the supply voltage, the bias voltage is between −1 and −2.5 V

Finally, to demonstrate the benefit of a small contact resistance for the dynamic TFT performance, 11-stage unipolar ring oscillators were fabricated on the same PEN substrates as the TFTs discussed above (Fig. 8). All TFTs in the ring oscillators have a channel length of 1 µm and a total gate-to-contact overlap of 10 µm. For this channel length and gate-to-contact overlap, the effective carrier mobilities are 1.3 cm^2 ^V^−1^ s^−1^ for the TC TFTs and 1.7 cm^2 ^V^−1^ s^−1^ for the BC TFTs (Supplementary Figure 5). The ring oscillators utilize the biased-load inverter design^61^. The signal-propagation delay (*τ*) is calculated from the oscillation frequency (*f*_*osc*_) and the number of stages (*n*) by *τ* = 1/(2*nf*_*osc*_)^62^. At a supply voltage of 3.7 V, the measured signal-propagation delay is 178 ns per stage for the ring oscillator based on the TC TFTs and 138 ns per stage for the ring oscillator based on the BC TFTs, confirming the effect of the contact resistance on the frequency behavior of the TFTs. These signal delays are to our knowledge the shortest delays reported to date for organic ring oscillators on flexible substrates^63^ and the shortest delays for organic ring oscillators on any substrate at a supply voltage of less than 50 V^64^.

## Discussion

Through an analysis of the contact resistance in TFTs with different gate-dielectric thicknesses, we have found strong experimental indications that it is possible to fabricate bottom-gate, bottom-contact organic TFTs that show lower contact resistance than comparable top-contact TFTs. As predicted by Zojer et al.^30^, we have found that for sufficiently small gate-dielectric thickness, bottom-contact TFTs have lower contact resistance than top-contact TFTs, so long as sufficient measures are taken to control the semiconductor thin-film morphology across the contact-channel interface and to minimize the barrier height at the contact-semiconductor interface. This was accomplished here by employing a thin hybrid gate dielectric composed of aluminum oxide passivated with an alkylphosphonic acid SAM in combination with PFBT-modified gold source and drain contacts in the bottom-contact TFTs. The potential of this approach to improve the static and dynamic performance of organic TFTs is most significantly exemplified here by bottom-contact DPh-DNTT TFTs with a gate-dielectric thickness of 5.3 nm fabricated on flexible PEN substrates which show a channel-width-normalized contact resistance as small as 29 Ωcm. In addition to a low contact resistance, bottom-contact TFTs can show improvements in other performance metrics, including subthreshold swings as small as 62–64 mV/decade and on/off current ratios as high as 10^9^. Furthermore, the lower contact resistance of the bottom-contact TFTs enables higher frequencies in flexible organic-TFT circuits operating at low voltages, as shown here by the signal-propagation delay of 138 ns per stage at a supply voltage of 3.7 V, obtained in 11-stage unipolar ring oscillators based on bottom-contact DPh-DNTT TFTs fabricated on flexible PEN substrates. It is possible that even lower contact resistance is achievable with other combinations of interface layers, gate dielectrics and semiconductors in the bottom-gate, bottom-contact architecture. Further reductions in contact resistance, ideally in combination with smaller lateral TFT dimensions, are then expected to yield even higher dynamic TFT performance^2^.

## Methods

### TFTs with different gate-oxide thicknesses on Si substrates

The TFTs that were used to study the relation between the gate-dielectric thickness and the contact resistance were fabricated on heavily doped silicon wafers (525 µm thickness). To reduce the effects of substrate-to-substrate variations, bottom-contact (BC) and top-contact (TC) TFTs with a common gate-dielectric thickness were fabricated on the same substrate in close proximity to each other (separated by about 100–200 µm). The silicon substrate serves as a global gate electrode for all TFTs on the substrate (Fig. 1a). As the first component of the gate dielectric, aluminum oxide (Al_2_O_3_) was deposited by atomic-layer deposition (ALD, Savannah 100, Cambridge NanoTech Inc.; substrate temperature 250 °C, 10 cycles/nm) with a thickness of 3, 30, 50, or 100 nm. The silicon wafers were then cut into strips (0.5 × 3 cm). The Al_2_O_3_ surface was activated by oxygen plasma (Oxford Instruments; oxygen flow rate 30 sccm, partial pressure 10 mTorr, plasma power 200 W, duration 30 s) and then passivated with a self-assembled monolayer (SAM) by immersing the substrate into a 1-mM solution of *n*-tetradecylphosphonic acid (PCI Synthesis, Newburyport, MA, U.S.A.) in 2-propanol (VLSI grade) for one to two hours^33^. Afterwards, the substrates were rinsed in 2-propanol and dried on a hotplate (150 °C, 1 min). The capacitance of these dielectrics was calculated assuming relative dielectric constants (*ϵ*_*r*_) of 9 for Al_2_O_3_ and 2.5 for the phosphonic acid SAM^33^. Next, gold bottom source and drain contacts were deposited by thermal evaporation in vacuum onto the surface of the Al_2_O_3_/SAM gate dielectric and modified with a monolayer of pentafluorobenzenethiol (PFBT, Santa Cruz Biotechnology, Heidelberg, Germany) by immersing the substrates into a 10-mM solution of PFBT in 2-propanol for 30 min. The substrates were then rinsed with 2-propanol. A 20-nm-thick layer of DPh-DNTT (Nippon Kayaku, kindly provided by Koichi Ikeda) was then deposited by sublimation in vacuum (base pressure 10^–6^ mbar, substrate temperature 90 °C, deposition rate 0.3 Å s^−1^) onto all four substrates simultaneously (in order to minimize substrate-to-substrate variations). Prior to electrical measurements, the unpatterned DPh-DNTT layer was scratched away around each set of source and drain contacts using a probe needle. Electrical measurements were then performed on the BC TFTs, followed by depositing the gold top source and drain contacts onto the organic semiconductor layer in close proximity to the bottom contacts and performing the electrical measurements on the TC TFTs. All contacts have an area of 200 × 200 µm and were patterned using a silicon stencil mask (IMS Chips, Stuttgart, Germany) with channel lengths ranging from 4 to 50 µm^38^.

### TFTs and ring oscillators on flexible PEN substrates

We fabricated bottom-contact and top-contact DPh-DNTT TFTs and 11-stage unipolar ring oscillators on 125-µm-thick flexible polyethylene naphthalate substrates (Teonex^®^ Q65 PEN; provided by William A. MacDonald, DuPont Teijin Films, Wilton, U.K.) using a set of four silicon stencil masks (IMS Chips, Stuttgart, Germany) to define interconnects, gate electrodes, source and drain contacts, and the organic semiconductor layer (Fig. 1b)^62,65^. Prior to fabrication, the PEN substrates were baked at a temperature of 100 °C for 1 h and cleaned with 2-propanol. In the first fabrication step, 30-nm-thick gold interconnects and probe pads were deposited through the first stencil mask. For the gate electrodes, a 30-nm-thick layer of aluminum was deposited through the second stencil mask. In the case of the TFTs discussed above that were used to evaluate the relation between the gate-dielectric thickness and the contact resistance, the gate oxide was deposited by atomic-layer deposition. ALD has the advantage that the oxide thickness can be easily scaled over a wide range. However, one issue with ALD is that the oxide is not easily deposited selectively, so that subtractive patterning is usually required to create access to the gate electrodes underneath the oxide. For the TFTs discussed above, this issue was avoided by contacting the gate electrode (i.e., the doped silicon substrate) from the backside of the substrate. For devices and circuits on plastic substrates this is not an option. Therefore, for the TFTs and ring oscillators on PEN, we used plasma oxidation (Oxford Instruments, 30 sccm oxygen, 10 mTorr, 200 W, 30 s) to form a thin aluminum oxide (AlO_x_) layer selectively on the patterned aluminum gate electrodes. The completed gate dielectric is a stack of the 3.6-nm-thick layer of AlO_x_ and an *n*-tetradecylphosphonic acid SAM, resulting in a total dielectric thickness of 5.3 nm and a unit-area capacitance of 0.7 µF cm^−2.^^37^. For all subsequent layers, the fabrication procedure was the same as described above for the TFTs on silicon substrates, with the exception that the semiconductor layer is patterned with a stencil mask. In the ring oscillators, the drive TFTs have channel widths of 80 µm and the bias TFTs have channel widths of 20 µm. In the two buffer inverters prior to the output node the drive TFT has a channel width of 220 µm and the bias TFT has a channel width of 60 µm.

### Semiconductor thin-film morphology characterization

The thin-film morphology of the DPh-DNTT layer was characterized using tapping-mode atomic force microscopy (AFM, Bruker Dimension Icon), scanning electron microscopy (SEM, Zeiss Merlin), and grazing incidence X-ray diffraction (GIXRD, Rigaku SmartLab). The X-ray diffractometer is equipped with a 9 kW copper source. The grazing incidence angle was set to 0.15°. The detector moved horizontally at 2θ = 0.15° and 2θχ from 15° to 30° in steps of 0.1°. The X-ray beam size was set to 5 × 0.1 mm. The diffraction peaks were assigned using the PDXL software with the standard Gaussian distribution method. SEM and AFM were performed on completely processed TFTs, while GIXRD required samples either without any metal or with the gold layer covering the entire substrate, due to the large spot size required for the measurement. Three silicon substrates were thus prepared for the GIXRD measurements, with 30-nm thick DPh-DNTT deposited onto 30-nm thick Au, 30-nm thick Au treated with PFBT, and atomic-layer-deposited Al_2_O_3_ passivated with an *n*-tetradecylphosphonic acid SAM.

### Electrical measurements

All electrical measurements, including the bias-stress measurements summarized in Supplementary Figure 6, were performed in ambient air at room temperature (292 K). The capacitance of the gate dielectrics, the current-voltage characteristics of the TFTs, and the signal-propagation delays of the ring oscillators were measured using an Agilent 4156 C Semiconductor Parameter Analyzer, a Tektronix TDS1000 oscilloscope, a Femto DPLCA-200 low-noise transimpedance amplifier, and gold-plated tungsten probe tips (EPP GmbH) with a tip radius of 50 µm for contacting the probe pads.

## Supplementary information

Supplementary Information

## Data Availability

The data that support the findings of this study are available from the corresponding author on reasonable request.
