# Supplementary material for: Small contact resistance and high-frequency operation of flexible low-voltage inverted coplanar organic transistors
Source: Nat Commun. 2019 Mar 8;10:1119. doi: 10.1038/s41467-019-09119-8 (PMC6408530; doi:10.1038/s41467-019-09119-8)
Supplement: Supplementary file 1 — Supplementary Information [file 41467_2019_9119_MOESM1_ESM.pdf]

Supplementary Information

Small contact resistance and high-frequency operation of flexible low-voltage inverted coplanar organic transistors

James W. Borchert, Boyu Peng, Florian Letzkus, Joachim N. Burghartz,
Paddy K. L. Chan, Karin Zojer, Sabine Ludwigs & Hagen Klauk

Supplementary Figures

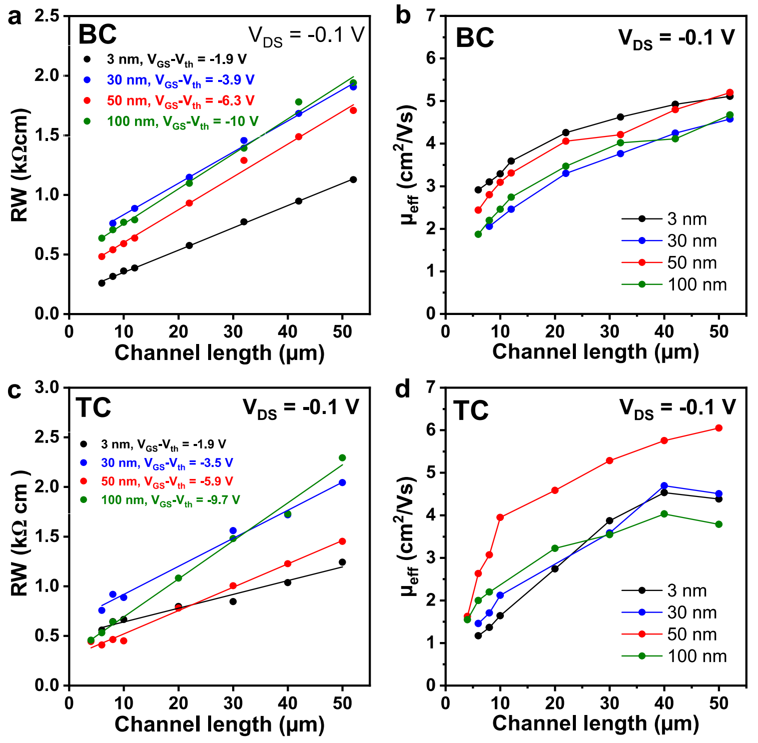

Figure 1: Analysis of DPh-DNTT TFTs with different gate-dielectric thicknesses. The bottom contact (BC) and top contact (TF) TFTs are fabricated on highly-doped cilicon substrates and have SAM-modified atomic-layer-deposited Al_2O_3 gate dielectrics with gate-oxide thicknesses of 3, 30, 50 and 100 nm, channel lengths ranging from 4 to 50 µm, and a channel width of 200 µm. (**a,c**) Transmission line method (TLM) analysis performed at the largest gate overdrive voltage for each gate-oxide thickness. All fits show $R^2 > 0.9$. (**b,d**) Effective carrier mobility plotted as a function of the channel length.

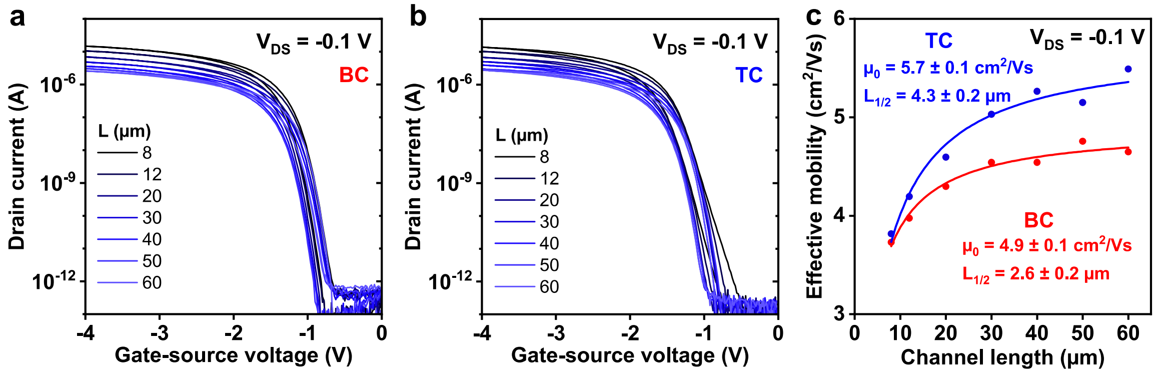

Figure 2: DPh-DNTT TFTs fabricated on flexible PEN substrates. The TFTs have channel lengths ranging from 8 to 60 µm, a channel width of 200 µm, and a total gate-to-contact overlap of 10 µm. (**a**) Transfer characteristics of the bottom-contact TFTs. (**b**) Transfer characteristics of the top-contact TFTs. The transfer data from **a** and **b** was employed for the TLM analysis reported in **Figure 5** and **Table 1** of the main manuscript. (**c**) Effective carrier mobility (μ_{eff}) plotted as a function of the channel length. The data are fit to the equation $\mu_{eff} = \mu_0/(1+L_{1/2}/L)$, where μ_0 is the intrinsic channel mobility, L is the channel length, and $L_{1/2}$ is the channel length at which μ_{eff} = ½ μ_0.

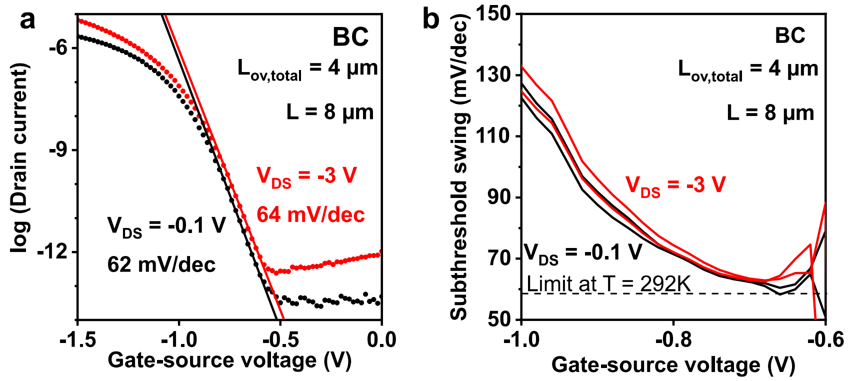

Figure 3: Subthreshold characteristics of bottom-contact DPh-DNTT TFTs fabricated on flexible PEN. The TFTs have a channel length of 4 µm, a channel width of 200 µm, and a total gate-to-contact overlap of 4 µm. (**a**) Exponential fit to the subthreshold region of the transfer characteristics of the TFT with the steepest subthreshold swing, yielding subthreshold swings of 62 and 64 mV/decade at drain-source voltages of -0.1 and -3 V, respectively. (**b**) Derivative of the measured transfer curves plotted as a function of the gate-source voltage. The dotted line denotes the theoretical limit of the subthreshold swing at a temperature of 292 K, i.e., 58 mV/decade.

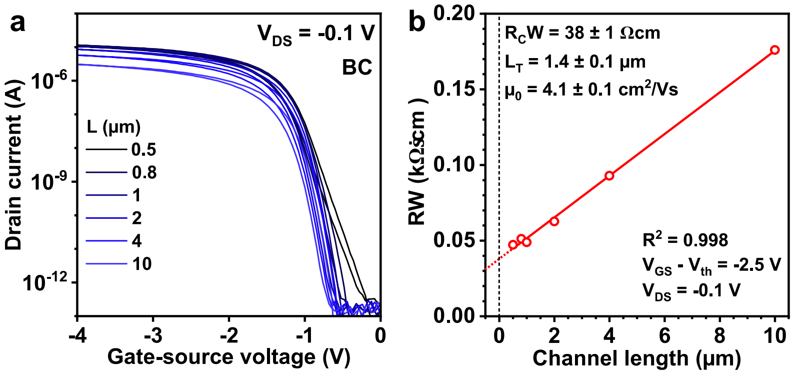

Figure 4: Bottom-contact DPh-DNTT TFTs with short channel lengths fabricated on a PEN substrate. (**a**) Measured transfer curves of the short-channel bottom-contact DPh-DNTT TFTs fabricated on flexible PEN substrates employed for the TLM analysis reported in **Figure 7** of the main manuscript. The TFTs have channel lengths ranging from 0.5 to 10 µm, a channel width of 50 µm, and a total contact overlap length of 10 µm. (**b**) TLM analysis performed at the largest gate overdrive voltage.

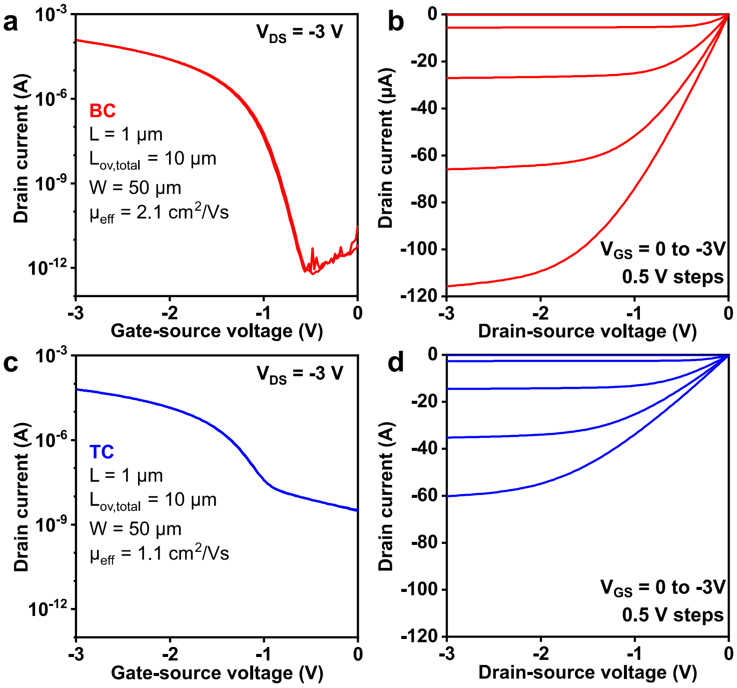

Figure5: DPh-DNTT TFTs with a channel length of 1 µm fabricated on PEN substrates. (**a,c**) Transfer and (**b,d**) output characteristics of bottom-contact and top-contact DPh-DNTT TFTs fabricated on flexible polyethylene naphthalate (PEN) substrates having a channel length of 1 µm, a channel width of 50 µm, and a total gate overlap length of 10 µm.

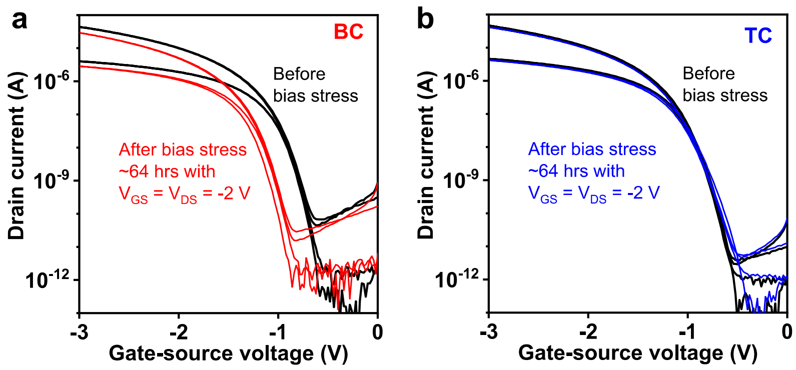

Figure 6: Bias-stress stability of DPh-DNTT TFTs fabricated on a silicon substrate. These TFTs were fabricated by depositing a 30-nm-thick aluminum gate electrode onto the doped silicon substrate and then forming a 3.6-nm-thick layer of aluminum oxide by oxygen-plasma growth and a 1.7-nm-thick *n*-tetradecylphosphonic acid self-assembled monolayer from solution to obtain a 5.4-nm-thick AlO_x/SAM gate dielectric. The TFTs have a channel length of 20 µm and a channel width of 200 µm. The bias stress was performed by applying gate-source and drain-source voltages of -2 V continuously for a duration of 64 hours in ambient air. The effective carrier mobility of the bottom-contact TFT (**a**) was 3.5 cm^2/Vs prior to and 3.0 cm^2/Vs after the bias-stress experiment. The effective mobility of the top-contact TFT (**b**) was 3.7 cm^2/Vs prior to and 3.5 cm^2/Vs after the bias-stress experiment.
